# Supplementary material for: Calcium Channel Blockers Are Associated with Nocturia in Men Aged 40 Years or Older
Source: J Clin Med. 2021 Apr 9;10(8):1603. doi: 10.3390/jcm10081603 (PMC8070101; doi:10.3390/jcm10081603)
Supplement: Supplementary file 1 [file jcm-10-01603-s001.pdf]

Supplementary Materials

Table S1. International Prostate Symptom Score (IPSS) questionnaire.

	**Not at all**	**Less than 1 time in 5**	**Less than half the time**	**About half the time**	**More than half the time**	**Almost always**	**Your score**
**1. Incomplete emptying**<br>Over the past month, how often have you had a sensation of not emptying your bladder completely after you finish urinating?	0	1	2	3	4	5	
**2. Frequency**<br>Over the past month, how often have you had to urinate again less than two hours after you finished urinating?	0	1	2	3	4	5	
**3. Intermittency**<br>Over the past month, how often have you found you stopped and started again several times when you urinated?	0	1	2	3	4	5	
**4. Urgency**<br>Over the last month, how difficult have you found it to postpone urination?	0	1	2	3	4	5	
**5. Weak stream**<br>Over the past month, how often have you had a weak urinary stream?	0	1	2	3	4	5	
**6. Straining**<br>Over the past month, how often have you had to push or strain to begin urination?	0	1	2	3	4	5	
	**None**	**1 time**	**2 times**	**3 times**	**4 times**	**5 times or more**	**Your score**
**7. Nocturia**<br>Over the past month, how many times did you most typically get up to urinate from the time you went to bed until the time you got up in the morning?	0	1	2	3	4	5	
	**Delighted**	**Pleased**	**Mostly satisfied**	**Mixed: Equally satisfied / dissatisfied**	**Mostly dissatisfied**	**Unhappy**	**Terrible**
**Quality of life due to urinary symptoms**<br>If you were to spend the rest of your life with your urinary condition the way it is now, how would you feel about that?	0	1	2	3	4	5	6

Table S2. Overactive bladder symptom score (OABSS) questionnaire.

Question	Frequency	Score
**1. Daytime frequency** How many times do you typically urinate from waking in the morning until sleeping at night?	7 or less	0
	8 to 14	1
	15 or more	2
**2. Nighttime frequency** How many times do you typically wake up to urinate from sleeping at night until waking in the morning?	0	0
	1	1
	2	2
	3 or more	3
**3. Urgency** How often do you have a sudden desire to urinate, which is difficult to defer?	Not at all	0
	Less than once a week	1
	Once a week or more	2
	About once a day	3
	2 to 4 times a day	4
	5 times a day or more	5
**4. Urgency incontinence** How often do you leak urine, because you cannot defer the sudden desire to urinate?	Not at all	0
	Less than once a week	1
	Once a week or more	2
	About once a day	3
	2 to 4 times a day	4
	5 times a day or more	5

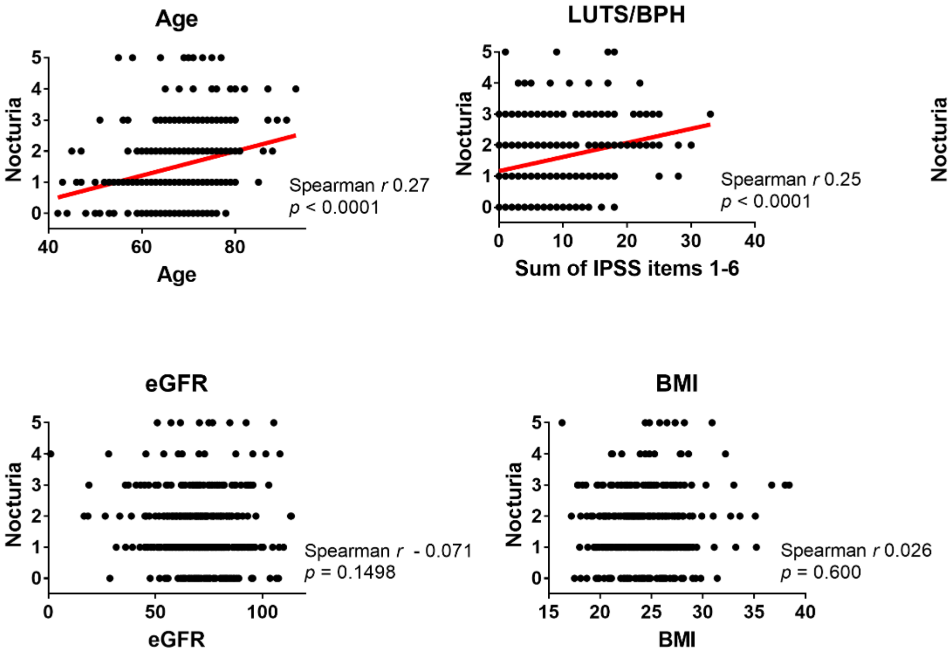

Figure S1. Correlations between nocturia and other covariates. Correlations between nocturia episodes and other covariates, including age, the sum of IPSS scores on items 1–6, glycated hemoglobin, eGFR, and BMI, are shown. LUTS: lower urinary tract symptoms, Hb: hemoglobin, eGFR: estimated glomerular filtration ratio, BMI: body mass index.

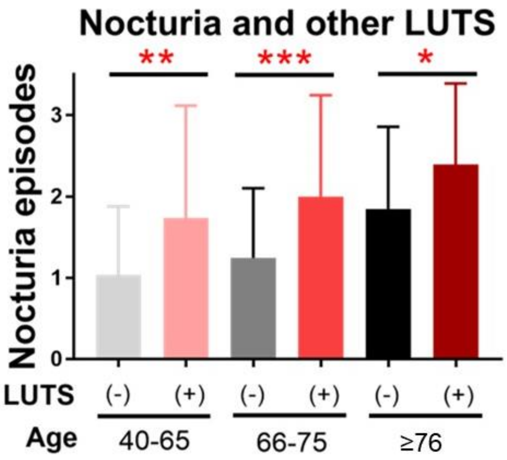

Figure S2. Patients with LUTS other than nocturia experienced more nocturia episodes in each age group.LUTS: lower urinary tract symptoms. LUTS (+) means that the sum of IPSS items 1–6 was more than 6. * $p < 0.05$, ** $p < 0.01$, *** $p < 0.001$.
